# Caregiver perspectives on patient-focused drug development for Phelan-McDermid syndrome

**DOI:** 10.1186/s13023-024-03141-w

**Published:** 2024-03-26

**Authors:** Luciana Gizzo, Geraldine Bliss, Chrystal Palaty, Alexander Kolevzon

**Affiliations:** 1 University of New England College of Osteopathic Medicine, Biddeford, ME USA; 2 CureSHANK, Beverly Hills, CA USA; 3 Metaphase Health Research Consulting Inc., Vancouver, Canada; 4 Seaver Autism Center for Research and Treatment, Department of Psychiatry, Icahn School of Medicine at Mount Sinai, One Gustave L. Levy Place, Box 1230, New York, NY 10029 USA

**Keywords:** Phelan-McDermid syndrome, PMS, SHANK3, Autism spectrum disorder, ASD, Drug development, Caregiver perspective

## Abstract

**Background:**

Phelan-McDermid syndrome (PMS) is a neurodevelopmental disorder caused by *SHANK3* haploinsufficiency with clinical manifestations that can be devastating and profoundly affect quality of life.

**Results:**

The Externally Led Patient-Focused Drug Development (EL-PFDD) meeting was an opportunity for families affected by PMS to share with the Food and Drug Administration (FDA) how symptoms impact their lives and how treatments could be most meaningful. The Voice of the Patient report serves as a summary of this meeting to influence upcoming drug development and clinical trials. The purpose of this report is to provide a clinical perspective on the results of the EL-PFDD meeting to amplify the voice of these caregivers to the scientific community.

**Conclusions:**

Caregivers prioritize an improved quality of life for their loved ones characterized by improved cognitive function, improved communication, increased independence, and reduced risk of regression. With these caregiver priorities in mind, this report provides the FDA and the scientific community with a clear understanding of which aspects of PMS should influence the development of future therapeutics.

## Background

### Phelan-McDermid syndrome overview

There are approximately 3,100 cases of Phelan-McDermid syndrome (PMS) identified worldwide by the Phelan-McDermid Syndrome Foundation (PMSF). PMS is caused by a terminal 22q13.3 deletion encompassing the *SHANK3* gene or a pathogenic sequence variant in *SHANK3,* both resulting in haploinsufficiency. The result is a clinically heterogeneous syndrome with manifestations that include intellectual disability (ID), autism spectrum disorder (ASD), sleep disturbance, seizures, hypotonia, gastrointestinal dysfunction, and a wide range of dysmorphic features, among many other characteristics [1, 2]. In addition, neuropsychiatric symptoms with features of bipolar disorder and catatonia are increasingly recognized, as well as the risk of regression and loss of skills [3, 4]. *SHANK3* deletions or sequence variants are among the most common causes of ASD, accounting for 0.5—2% of ASD cases [5–7]. Data from genotype–phenotype studies are now emerging [8, 9], and in addition to results from ongoing natural history studies, may eventually help predict disease course to guide caregivers and clinicians regarding future expectations of quality of life.

### Current medication therapies: advancements and barriers

Sleep, cognition, language, and behavioral disturbances are cited as the most challenging aspects of PMS [10] and therefore have become a primary focus in the field of treatment development. Several treatment studies suggest promise but require replication on a larger scale. Both insulin-like growth factor-1 (IGF-1) and the closely related human growth hormone have been shown in small trials to improve symptoms of social withdrawal, sensory sensitivity, and hyperactivity in children with PMS [11–14]. In addition, Neuren Pharmaceuticals recently completed recruitment for a Phase 2 trial of NNZ-2591, an analog of IGF-1, in children with PMS. Other small trials or case reports have described benefit with Q10 ubiquinol [15], intranasal insulin [16], lithium [17], low dose risperidone [18], and intravenous immunoglobulin (IVIG) [19]. Gene therapy approaches are also being developed, including using an adeno-associated virus (AAV) vector to deliver *SHANK3* gene directly to the central nervous system [20] and RNA-based technology to increase expression of *SHANK3* from the intact allele [21].

Despite a growing abundance of research advancing potential treatments in PMS, significant barriers persist. A clinical outcome assessment appropriate for people with PMS and profound language impairment and intellectual disabilities has yet to be validated for use in clinical trials. However, several measures show promise, including one developed by Neuren Pharmaceuticals to capture clinical global improvement, and another developed at the Seaver Autism Center for Research and Treatment at Mount Sinai to assess sensory reactivity symptoms called the Sensory Assessment for Neurodevelopmental Disorders (SAND) [22]. Improved clinical outcome measures, along with the development of biomarkers using electrophysiology or eye tracking technologies, are a major priority for the field and may pave the way for successful large-scale clinical trials in the future.

### Meeting overview and key themes

The purpose of the Externally Led Patient-Focused Drug Development (EL-PFDD) in PMS was to describe the varied manifestations of the syndrome and to more precisely direct treatment development toward improving quality of life for individuals with PMS. The EL-PFDD in PMS implemented a systematic approach to collecting data on caregiver perspectives on the condition and available treatments with the ultimate goal of informing regulatory agencies and facilitating the process of drug development.

## Results

### Demographic information

Prior to obtaining data for each session of the EL-PFDD, demographic information was collected from 228 meeting participants. Most participants were caring for a loved one with a 22q13 terminal deletion (63%), 34% had *SHANK3* sequence variants, and 3% did not know the type of genetic change. The majority of participants resided in the United States Eastern Time Zone (43%). The sex of the individuals with PMS was evenly distributed between males (52%) and females (48%). The mean age of affected individuals was 14.9 years old (range 1–60; SD = 13.1) and the mean age of diagnosis was 7 years old (range 0–40; SD = 8.3).

### Session one: living with PMS: symptoms and daily impact

**Fig. 1:**
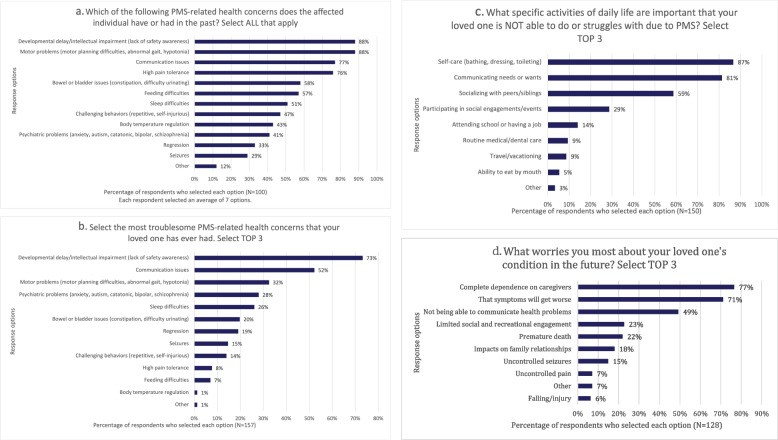
Session one poll results. **a**-**d** represent the poll results of Session 1 of the EL-PFDD meeting which addressed the topic “*Living with PMS: Symptoms and Daily Impact”.* Data are reported as percentages based on the number of participants who selected each multiple choice response option, out of the total number of unique participants in each poll. **a** Past PMS-related health concerns. **b** Current PMS-related health concerns. **c** Challenges in activities of daily living in PMS. **d** Worrisome future outcomes for individuals with PMS

The most commonly reported health concerns among participants included developmental delays and motor problems (motor planning difficulties, hypotonia, and abnormal gait) followed by communication difficulties and sensory hyporeactivity. Other common symptoms included bowel or bladder dysregulation and feeding and sleeping difficulties (Fig. 1a). Of the multitude of health concerns in PMS, developmental delay/intellectual disability, communication issues, and motor problems were noted as most troublesome overall (Fig. 1b). Activities of daily life that were reported to be most impacted in PMS included self-care, such as bathing, dressing and toileting. Communicating needs as well as social communication were also reported to be significantly impacted in PMS (Fig. 1c). When asked what worries caregivers most, concerns about the future, prognosis, the risk of total dependence on caregivers, and the inability to communicate were more concerning than physical health problems such as uncontrolled seizures or pain (Fig. 1d).

### Session two: PMS current and future treatments

**Fig. 2:**
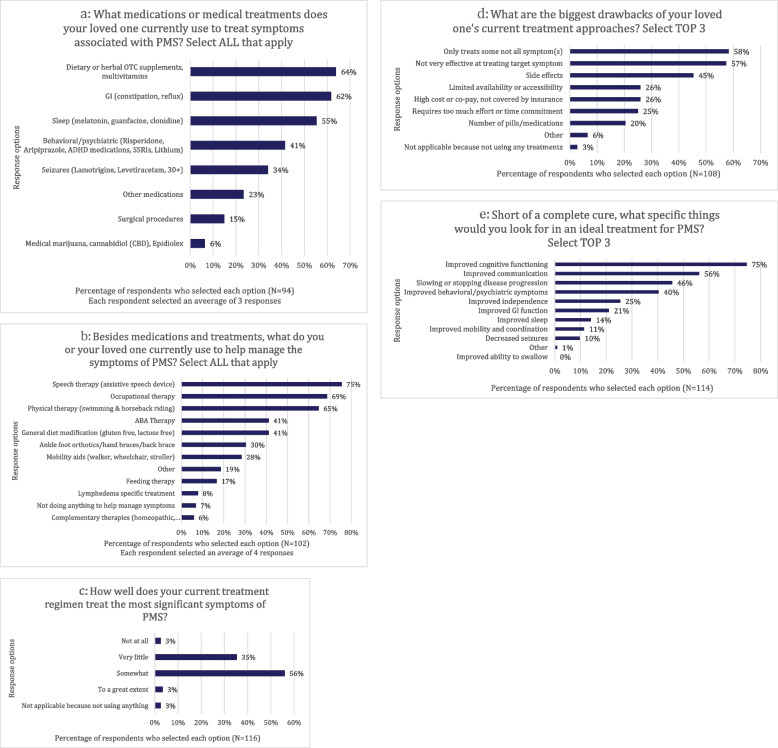
Session Two Poll Results. **a**-**e** represent the poll results of Session 2 of the EL-PFDD meeting which addressed “*PMS Current and Future Treatments”.* Data are reported as percentages based on the number of participants who selected each multiple choice response option, out of the total number of unique participants in each poll. **a** Use of medical treatments in PMS. **b** Use of non-medical therapies in PMS. **c** Effectiveness of current PMS treatment. **d** Limitations of current PMS treatment. **e** Potential treatment targets for PMS

The most frequently used medications included dietary/herbal supplements or vitamins, gastrointestinal agents, and sleep therapeutics (Fig. 2a). In addition to medications, speech, occupational and physical therapy were most commonly used (Fig. 2b). Fifty-six percent of caregivers reported that their current treatment regimen only somewhat addressed the most significant symptoms of PMS (Fig. 2c). The biggest drawbacks to current treatment approaches included the inability to address all symptoms of PMS, followed by ineffectiveness at treating the target symptom, and then adverse effects (Fig. 2d). When asked what an ideal treatment for PMS would entail, 75% of caregivers prioritized cognitive functioning, 56% advocated for improved communication, and 46% noted slowing or stopping symptom progression (Fig. 2e). Psychiatric symptom management was most important for 40% of participants, gastrointestinal symptom management for 21%, and improved sleep for 14% (Fig. 2e).

## Discussion

### Voices of caregivers and patient perspectives

Overall, the results of the EL-PFDD meeting provide evidence of the profound challenges posed by PMS, for both affected individuals and their caregivers. PMS is characterized by a wide range of disabilities and the severity of symptoms likewise varies greatly across individuals. Despite the heterogeneous constellation of symptoms, specific issues repeatedly emerged throughout the meeting as the most debilitating. Specifically, developmental delay, intellectual disability, and the inability to communicate effectively were consistently reported as the most common and the most burdensome for caregivers. These reports were replicated when caregivers were asked which aspects of the disease are most worrisome. Impairments can be further exacerbated by regression, most commonly in the communication domain. Communicating needs, self-care (e.g., bathing, dressing, toileting) and socializing with peers/siblings are also greatly impacted in PMS. Daily living skills, loss of communication, and worsening disease progression remained high priorities for treatment, in addition to treatment for common physical health related concerns such as increased pain tolerance, seizures, hypotonia, and motor planning difficulties. Our results are consistent with a published study that includes worldwide surveys that corroborate both our genetic demographics and caregiver concerns [23]. According to this study, PMS is more frequently caused by a deletion in chromosome 22q13.3 than a SHANK3 gene variant, consistent with our findings (63% to 34%, respectively). Additionally, this paper stated that the most common issues were speech/communication, learning disabilities, intellectual disability, and behavior, which closely mirrors our findings that communication, cognitive function, and reducing the risk of regression were the top caregiver priorities. These findings emphasize the importance caregivers place on improving quality of life for their loved ones. Given this theme, treatment development should focus on clinical trials with interventions that can improve quality of life. Ideally, the future treatment landscape should include interventions that can potentially address cognition and communication and promote greater independence for affected individuals.

### Development of clinically impactful treatment/patient focused drug development:

Despite debilitating manifestations, there are no treatments for PMS currently approved by the Food and Drug Administration (FDA). Therefore, caregivers remain especially concerned for the future as their loved ones age. While most individuals with PMS start receiving therapeutic interventions (e.g., speech, physical and occupational therapies) early in childhood and continue through adolescence, existing therapies are only partially effective and typically only address selected symptoms. Medication adverse effects and regression also further undermine potential benefits achieved from therapy. Anti-epileptic treatments in particular were often cited as having profound adverse effects, complicating reports that seizures present a significant burden for both individuals with PMS and caregivers. In addition, while adverse effects, cost of treatment, and insurance coverage were identified as barriers to care, lack of effective treatments were ranked higher as a concern. The risk of adverse effects and costs must nevertheless be carefully balanced in treatment development. Further, route of medication administration (e.g., oral, injection, intranasal) should also be considered in terms of ease of use and feasibility for caregivers, particularly when used under rescue, or acute circumstances. Overall, treatment development is an area of huge unmet need in PMS and the field should prioritize discovery of targeted therapies that address caregivers’ areas of concern. Patient quality of life, cognitive ability, and communication remain the greatest concerns for caregivers and should therefore be the focus of treatment development. Findings from this meeting may help inform the development of PMS-specific clinically meaningful endpoints for current and future clinical trials, as well as encourage additional researchers and industry to investigate new treatments.

An inherent limitation of this data is that it was collected under true “real world” conditions. Poll response variability was most likely due to personal choice and/or opportunity. Online polling was voluntary, so participants could choose to answer the questions most relevant to them. Additionally, the EL-PFFD meeting took place in one day across several hours, with poll questions offered during discrete time points. Therefore, it is likely that many EL-PFDD meeting participants were attending the meeting and responding to poll questions while simultaneously executing daily caregiving duties, which may have impacted their ability to participate in a consistent and timely manner.

## Conclusions

Individuals with PMS often have a complex and interrelated constellation of symptoms with few effective treatment options. CureSHANK and the PMSF hosted the PMS EL-PFDD meeting to provide a patient and caregiver perspective of the symptoms and burden associated with PMS in daily life, as well as to highlight the massive unmet treatment needs experienced by families. Results from this meeting serve to provide the FDA and the scientific community with data to promote patient-focused drug development in PMS. Overall, caregivers who attended the EL-PFDD meeting urged the prioritization of targeted therapeutics that improve cognitive function, communication, and reducing the risk of regression. Seizures, sleep changes, hypotonia, motor planning difficulties, and sensory hyporeactivity were also significant priority areas for caregivers. The EL-PFDD and this report serve to amplify the needs of individuals with PMS and to promote the development of treatments that improve independence and enhance overall quality of life for individuals with PMS.

## Methods

The EL-PFDD meeting for PMS was held on November 8, 2022 via live stream on CureSHANK and YouTube. A welcome and overview was conducted by Geraldine Bliss, President and Co-Founder of CureSHANK, followed by opening remarks from Dr. Wilson Bryan of the FDA. A discussion on the background of PMS was provided by Dr. Alexander Kolevzon, Clinical Director of the Seaver Autism Center for Research and Treatment at the Icahn School of Medicine at Mount Sinai. After an introduction and meeting overview given by James Valentine, JD, MHS, of Hyman, Phelps and McNamara and co-moderator, audience polling was conducted. Session 1 (“Phelan-McDermid Syndrome Patient Voices: Symptoms and Daily Impacts”) included panelist presentations where caregivers provided personalized stories regarding the symptomatology and daily manifestations of PMS. Audience members then participated in Poll Everywhere sessions regarding which symptoms of PMS were most concerning to caregivers. Session 2 (“Phelan-McDermid Syndrome Patient Voices: Current and Future Treatments”) included panelist presentations amplifying which aspects of PMS should warrant future drug development and the benefits and challenges of current treatment modalities. These issues were then queried with using Poll Everywhere software from audience members. Finally, a summary of the meeting was presented by Larry Bauer RN, MA, of Hyman, Phelps & McNamara, P.C, and consulting partner, and future directions were outlined by Dr. Kate Still, Scientific Director of the PMSF. In the months following the meeting, Chrystal Palaty, a scientific writer, composed a Voice of the Patient (VoP) Report [24] which provides an organized compilation of all caregiver stories that were either submitted online or discussed throughout the live meeting. The VoP Report, along with this review, are intended to direct the scientific community and the FDA regarding future drug development and management of patients with PMS.

### Statistical analysis

Descriptive statistics from Poll Everywhere data were performed using Microsoft Excel. Demographic results (Table 1) were calculated using the total number of participants for that poll question as the denominator and participants were instructed to select all options that applied. For the data obtained during Session 1 and Session 2, poll results are presented as a percentage of unique participants to account for the fact that for most poll questions participants were allowed to select multiple options. We selected this method for presenting the data to most accurately reflect the extent of responses.

**Table 1:** Demographics

Response Options	Number of Responses	Percentage (%)
**1a: Genetic change causing PMS** *(n* = *108)*		
A. Deletion	68	62.96
B. *SHANK3* variant	37	34.26
C. Not sure	3	2.78
**1b: Residence ***(n* = *114)*		
A. US Pacific time zone	19	16.67
B. US Mountain time zone	6	5.26
C. US Central time zone	18	15.79
D. US Eastern time zone	49	42.98
E. US Alaska or Hawaii time zone	0	0
F. Europe/UK	19	16.67
G. Middle East	0	0
H. Asia	0	0
I. Canada	3	2.63
J. Brazil	0	0
K. Mexico	0	0
L. Africa	0	0
M. Other	0	0
**1c: Biological sex of affected individual** *(n* = *116)*		
A. Female	56	48.28
B. Male	60	51.72
C. Other	0	0
**1d: Age of affected individual** *(n* = *129)*		
A. Under 12 months	0	0
B. 1–2 years of age	7	5.43
C. 3–4 years of age	6	4.65
D. 5–10 years of age	46	35.66
E. 11–20 years of age	37	28.68
F. 21–30 years of age	21	16.28
G. 31–40 years of age	11	8.53
H. 41–60 years of age	1	0.78
I. Older than 60 years	0	0
**1e: Age of diagnosis** *(n* = *146)*		
A. 0–6 months	6	4.11
B. 7–12 months	13	8.9
C. 13–24 months	25	17.12
D. 25–36 months	26	17.81
E. 4–5 years	24	16.44
F. 6–10 years	17	11.64
G. 11–20 years	22	15.07
H. 21–30 years	7	4.79
I. 31–40 years	6	4.11
J. 41–60 years	0	0
K. Unsure	0	0

## Data Availability

The data and material for this publication was obtained at the November 8, 2022 EL-PFDD meeting and is the property of CureSHANK. The datasets used and/or analyzed during the current study are available from the corresponding author on request. The Voice of the Patient report is publicly available [24].

## Abbreviations

AAV9: Adeno-associated virus 9
ASD: Autism spectrum disorder
EL-PFDD: Externally Led Patient-Focused Drug Development
FDA: US Food and Drug Administration
ID: Intellectual disability
IGF-1: Insulin-like growth factor-1
IVIG: Intravenous immunoglobulin
PMS: Phelan-McDermid syndrome
PMSF: Phelan-McDermid Syndrome Foundation
SAND: Sensory Assessment for Neurodevelopmental Disorders

## References

[1] Soorya L, Kolevzon A, Zweifach J, Lim T, Dobry Y, Schwartz L (2013). Prospective investigation of autism and genotype-phenotype correlations in 22q13 deletion syndrome and SHANK3 deficiency. Mol Autism.

[2] Phelan K, Rogers RC, Boccuto L, Adam MP, Mirzaa GM, Pagon RA, Wallace SE, Bean LJH, Gripp KW (1993). Phelan-McDermid Syndrome. GeneReviews(®).

[3] Kohlenberg TM, Trelles MP, McLarney B, Betancur C, Thurm A, Kolevzon A (2020). Psychiatric illness and regression in individuals with Phelan-McDermid syndrome. J Neurodev Disord.

[4] Kolevzon A, Delaby E, Berry-Kravis E, Buxbaum JD, Betancur C (2019). Neuropsychiatric decompensation in adolescents and adults with Phelan-McDermid syndrome: a systematic review of the literature. Mol Autism.

[5] Durand CM, Betancur C, Boeckers TM, Bockmann J, Chaste P, Fauchereau F (2007). Mutations in the gene encoding the synaptic scaffolding protein SHANK3 are associated with autism spectrum disorders. Nat Genet.

[6] Leblond CS, Nava C, Polge A, Gauthier J, Huguet G, Lumbroso S (2014). Meta-analysis of SHANK mutations in autism spectrum disorders: a gradient of severity in cognitive impairments. PLoS Genet.

[7] Satterstrom FK, Kosmicki JA, Wang J, Breen MS, De Rubeis S, An JY (2020). Large-scale exome sequencing study implicates both developmental and functional changes in the neurobiology of autism. Cell.

[8] Sarasua SM, Boccuto L, Sharp JL, Dwivedi A, Chen CF, Rollins JD (2014). Clinical and genomic evaluation of 201 patients with Phelan-McDermid syndrome. Hum Genet.

[9] Levy T, Foss-Feig JH, Betancur C, Siper PM, Trelles-Thorne MDP, Halpern D (2022). Strong evidence for genotype-phenotype correlations in Phelan-McDermid syndrome: results from the developmental synaptopathies consortium. Hum Mol Genet.

[10] Bro D, O’Hara R, Primeau M, Hanson-Kahn A, Hallmayer J, Bernstein JA (2017). Sleep disturbances in individuals with Phelan-McDermid Syndrome: correlation with caregivers’ sleep quality and daytime functioning. Sleep.

[11] Kolevzon A, Bush L, Wang AT, Halpern D, Frank Y, Grodberg D (2014). A pilot controlled trial of insulin-like growth factor-1 in children with Phelan-McDermid syndrome. Mol Autism.

[12] Kolevzon A, Breen MS, Siper PM, Halpern D, Frank Y, Rieger H (2022). Clinical trial of insulin-like growth factor-1 in Phelan-McDermid syndrome. Mol Autism.

[13] Li T, Xie R, Zhao J, Xu H, Cui Y, Sun C (2022). Effectiveness of recombinant human growth hormone therapy for children with Phelan-McDermid Syndrome: an open-label, cross-over, preliminary study. Front Psychiatry.

[14] Sethuram S, Levy T, Foss-Feig J, Halpern D, Sandin S, Siper PM (2022). A proof-of-concept study of growth hormone in children with Phelan-McDermid syndrome. Mol Autism.

[15] Cucinotta F, Ricciardello A, Turriziani L, Mancini A, Keller R, Sacco R (2022). Efficacy and safety of Q10 ubiquinol with vitamins B and E in neurodevelopmental disorders: a retrospective chart review. Front Psychiatry.

[16] Zwanenburg RJ, Bocca G, Ruiter SA, Dillingh JH, Flapper BC, van den Heuvel ER (2016). Is there an effect of intranasal insulin on development and behaviour in Phelan-McDermid syndrome? A randomized, double-blind, placebo-controlled trial. Eur J Hum Genet.

[17] Serret S, Thümmler S, Dor E, Vesperini S, Santos A, Askenazy F (2015). Lithium as a rescue therapy for regression and catatonia features in two SHANK3 patients with autism spectrum disorder: case reports. BMC Psychiatry.

[18] Pasini A, D'Agati E, Casarelli L, Curatolo P (2010). Dose-dependent effect of risperidone treatment in a case of 22q13.3 deletion syndrome. Brain Dev..

[19] Bey AL, Gorman MP, Gallentine W, Kohlenberg TM, Frankovich J, Jiang YH (2020). Subacute neuropsychiatric syndrome in girls With SHANK3 mutations responds to immunomodulation. Pediatrics.

[20] Jaguar gene therapy launches with mission to accelerate breakthroughs in gene therapy for patients suffering from severe genetic diseases. Lake Forest. 2021. https://jaguargenetherapy.com/press-release/jaguar-gene-therapy-launches-with-mission-to-accelerate-breakthroughs-in-gene-therapy-for-patients-suffering-from-severe-genetic-diseases/. Accessed 25 Aug 2023. [press release].

[21] New Central Nervous System (CNS). Drug program added tp PYC’S pipeline. 2022. https://pyctx.com/wp-content/uploads/2022/09/New-Central-Nervous-System-Drug-Program-Added-to-PYCs-Pipeline.pdf. Last Accessed 8 Sept 2023. Published Online. [press release].

[22] Siper PM, Kolevzon A, Wang AT, Buxbaum JD, Tavassoli T (2017). A clinician-administered observation and corresponding caregiver interview capturing DSM-5 sensory reactivity symptoms in children with ASD. Autism Res.

[23] Landlust AM, Koza SA, Carbin M, Walinga M, Robert S, Cooke J (2023). Parental perspectives on Phelan-McDermid syndrome: Results of a worldwide survey. Eur J Med Genet.

[24] Bliss G, Palaty C. Phelan-McDermid syndrome voice of the patient report. Beverly Hills, California: CureShank, PMSF; 2023. https://www.cureshank.org/_files/ugd/91238e_3523a24967614b28b5bf82ea613133c4.pdf. Accessed 25 Aug 2023.

